# Treatment of Pediatric Acute-Onset Neuropsychiatric Disorder in a Large Survey Population

**DOI:** 10.1089/cap.2017.0101

**Published:** 2018-03-01

**Authors:** Denise Calaprice, Janice Tona, Tanya K. Murphy

**Affiliations:** ^1^ Calaprice-Whitty Consulting, Inc., Lake Arrowhead, California.; ^2^ Department of Rehabilitation Science, University at Buffalo, Buffalo, New York.; ^3^ Department of Pediatrics, University of South Florida, St. Petersburg, Florida.; ^4^ Department of Psychiatry, University of South Florida, St. Petersburg, Florida.

**Keywords:** obsessive–compulsive disorder, survey, pediatric acute-onset neuropsychiatric syndrome, treatment

## Abstract

***Objective:*** The goal of this study was to investigate treatment histories and outcomes in a large community sample of youth with Pediatric Acute-onset Neuropsychiatric Syndrome (PANS), and, where appropriate, to examine the impact of immune deficiency on treatment outcomes.

***Methods:*** A comprehensive internet-based survey was completed by parents or guardians of youth who had received physician diagnoses of PANS, or by young adults (age 18+) who had themselves been diagnosed by a physician (*N* = 698). Data regarding the treatment histories of these patients, including the variety of medical and psychological treatments employed and the caregiver- or self-reported response to each, are presented.

***Results:*** The PANS patients in this study had commonly been treated with antibiotic (*N* = 675), anti-inflammatory (*N* = 437), and/or psychotropic therapy (*N* = 378). Response to antibiotic treatment was best when treatment was relatively aggressive, with broad-spectrum antibiotics and courses of >30 days generally producing the best results (i.e., up to 52% of patients achieving a “very effective” response). For immune-deficient patients (caregiver-reported laboratory studies below normal limits; *N* = 108), use of broad-spectrum antibiotics appeared to be particularly desirable. Anti-inflammatory therapies, including over-the-counter medications such as ibuprofen, were at least “somewhat effective” for most patients. Intravenous immunoglobulin (IVIG) had been used to treat PANS in 193 (28%) of the patients and was at least “somewhat effective” for 89%, although for 18% of these, the effect was not sustained. The highest rate of sustained response to IVIG treatment was seen in immune-deficient patients who received doses of at least 0.8 g/kg IVIG on a regular basis. Psychotropic medications, most commonly SSRIs (38% reported a trial), were commonly employed, but were often ineffective (e.g., 44% found SSRIs “somewhat” to “very effective”). Many patients (*N* = 473) had received some form of psychotherapy with some benefit, with cognitive behavioral therapy found to be at least somewhat effective in a majority of those treated with this modality.

***Conclusion:*** Among the PANS patients represented in this study, relatively aggressive treatment courses targeted at eradicating infection and modulating the inflammatory response appeared to provide the best caregiver-reported therapeutic results, and to be generally well tolerated. Given its relative efficacy and tolerability, treatment targeting the inflammatory response may represent an underutilized approach in this population. The results of this study should be considered in light of the limitations inherent in a self-selected and administered online survey.

## Introduction

The diagnostic criteria for Pediatric Autoimmune Neuropsychiatric Disorder Associated with Streptococcus (PANDAS) formulated by Swedo et al. in the late 1990's (Swedo et al. 1998) describe an acute-onset presentation of neuropsychiatric symptoms that is temporally related to a group A streptococcal (GAS) infection. Core symptoms include manifestations of obsessive–compulsive disorder (OCD) and/or new or worsening tics, whereas ancillary components may include attention-deficit/hyperactivity disorder (ADHD) symptoms, handwriting deterioration, choreiform movements, mood lability, anxiety, enuresis, and increased urinary frequency, among others. A broader variation of this disorder is PANS (Pediatric Acute-onset Neuropsychiatric Syndrome), which diagnostically requires acute-onset OCD or food refusal, most often but not necessarily in association with GAS or non-GAS infections, plus ancillary symptoms such as those described above for PANDAS (Swedo et al. 2012). The course of PANS is described as “sawtooth,” in that symptoms may remit between acute episodes, but patients often fail to return to baseline functioning even during remission. Similar to Sydenham's chorea, on which the original PANDAS model was based (Swedo 1994), PANS is presumed to have an autoimmune pathophysiology, and most PANS patients exhibit additional immune abnormalities, including comorbid autoimmune or inflammatory conditions and/or below normal or low-normal levels of immunoglobulins, in addition to having neuroactive autoantibodies (Frankovich et al. 2015; Nadeau et al. 2015).

Despite the pronounced level of suffering and disability often associated with PANS episodes (Frankovich et al. 2015; Nadeau et al. 2015; Calaprice et al. 2017; Tona et al. 2017), a gold-standard treatment strategy has yet to be established, and approaches vary greatly both in nature and effectiveness. The various treatment strategies employed focus on elimination of inciting infectious agents (antibiotics), support of the immune system (intravenous immunoglobulin, IVIG), suppression of the autoimmune mechanism (IVIG, plasmapheresis, steroids), and/or direct treatment of psychiatric symptoms (antidepressants, anxiolytics, mood stabilizers). However, all studies conducted on these treatments to date have involved small patient samples, most often limited to those seen at a single practice or research site, and a very limited number of specific (e.g., dosing) regimens. Associations between efficacy and patient heterogeneity (e.g., in particular with respect to immune competence) have also not been adequately studied.

With respect to treatment strategies involving antibiotics, remissions with antibiotic therapy have been anecdotally reported, and have been described in studies when therapy is applied during active infection (Murphy and Pichichero 2002; Falcini et al. 2013). Penicillin, cephalosporin, and amoxicillin, which are approved treatments for GAS infections (Shulman et al. 2012), have shown efficacy in the treatment of OCD and tic exacerbations in children with PANDAS (Murphy and Pichichero 2002; Falcini et al. 2013), and a recent study found some benefit of cefdinir in new-onset tics and OCD (Nadeau et al. 2015). Azithromycin in a randomized clinical trial has shown benefit for improving OCD in acute PANS presentations (Murphy et al. 2017). Prophylactic treatment with each of azithromycin and penicillin has demonstrated efficacy in one study (Snider et al. 2005), but another prophylaxis trial with penicillin failed to find a difference between penicillin and placebo (Garvey et al. 1999).

In small studies and individually reported cases, immunosupportive and autoimmune-suppressive strategies have also shown benefit in some patients. IVIG and plasmapheresis have shown benefit in an open-label trial of childhood infection-triggered OCD and tic disorders (Perlmutter et al. 1999), as well as in case reports (Allen et al. 1995; Elia et al. 2005; Latimer et al. 2015). Of note, IVIG has not been effective in adult patients with non-PANDAS tics (Hoekstra et al. 2004), and plasmapheresis has not been beneficial in non-PANDAS OCD (Nicolson et al. 2000). Case reports of steroids resulting in symptom remittance in Tourette's syndrome also exist (Matarazzo 1992; Allen et al. 1995), but these reports predate the identification of PANDAS or PANS, and the individuals in these reports also had recent infections, indicating the individuals may have had PANS and not Tourette's syndrome.

Although treatment with standard interventions, including psychotropic medications, may seem logical given PANS symptomatology, studies of such approaches are lacking, and there is some evidence that PANS youth respond to such therapies in an atypical manner. For example, youth with PANDAS appear to be at elevated risk for behavioral activation at typical doses of SSRIs, although some may gain therapeutic benefit from lower doses (Murphy et al. 2006). Cognitive behavioral therapy (CBT) has been found helpful to some youth with PANDAS or PANS (Storch et al. 2007; Nadeau et al. 2015), but larger controlled studies are needed to better understand the appropriate timing for and efficacy of these approaches, as well.

Treatment with complementary and alternative medicine (CAM) therapies have been anecdotally reported among PANS patients in social media, but no published studies to date have reported on the frequency or types of CAM therapies among PANS patients. CAM therapies, including dietary supplements and homeopathy, are reported to be used among patients with other autoimmune disorders, including multiple sclerosis (Stoll et al. 2012), and lupus (Haija and Schultz 2011).

In this study, we used data obtained from an online survey of a large, community-based sample of PANS patients (*N* = 698) to examine the range of treatments received in a naturalistic setting, the caregiver-reported (for minors) or self-reported (for adult patients) outcomes of these treatments, and the variability in these outcomes by the level of immunocompetence of the patient. This study was part of a broader survey of PANS patients with multiple research objectives, the objectives and results of which have been described elsewhere (Calaprice et al. 2017).

## Methods

We used a retrospective online survey to gather data regarding medical and family history, PANS symptomatology, and medical and non-medical interventions and outcomes for PANS. The 146-question survey was designed to be completed by a caregiver of at least one child diagnosed as having PANS, PANDAS, or PITAND, or by an adult PANS patient. To ensure that the questions were not only clinically meaningful but also constructed so as to maximize correct understanding by the intended population, the survey was developed through a collaborative effort by a clinical research consultant with expertise in survey design and patient-reported outcomes (D.C.), a pediatric psychiatrist with expertise in PANS (T.K.M.), and an occupational therapist with expertise in PANS (J.T.), and was reviewed by a PANS parent advocate with no clinical or scientific training and an epidemiologist, in addition to the IRB. To simplify completion, the survey used conditional logic so that questions that were logically irrelevant based on the participant's answers to previous questions were omitted from the participant's view of the survey. To standardize, as much as possible, the manner in which participants responded, response guidelines were provided for key multiple-choice questions. Approximately 20–40 minutes were required for survey completion.

Following approval of the study protocol, the survey instrument, and the electronic informed consent form by the Social and Behavioral Sciences Institutional Review Board (SBSIRB) at the University at Buffalo, a description of the study with a link to the survey, which was hosted at Vovici (www.vovici.com), was posted on the websites of the PANDAS Network (http://pandasnetwork.org) and the International Obsessive–Compulsive Disorder Foundation (IOCDF; www.ocfoundation.org). Then, email invitations were sent to families in the contact databases for these organizations, and information about the survey was included in monthly e-mail newsletters. The survey was also discussed in venues such as the Northeastern PANDAS/PANS Parent conference, and an internet-based radio show (RadioPandas). Finally, postcards and posters describing the study were sent to healthcare practices known to service PANS patients. Data collection concluded in 2014.

Clicking the survey link directed potential participants to a consent form detailing the purpose and procedures of the study. Contact information for study staff was provided so that questions could be answered before the decision to participate. To participate in the study, respondents had to certify that they were at least 18 years of age and either the parents or legal guardians of children who had been diagnosed by a physician with PANS, PANDAS, or PITAND, or had been diagnosed by a physician with PANS, PANDAS, or PITAND themselves. No documentation was examined to confirm the presence of key criteria; however, the following definition of and information about the disorder was offered as part of the survey introduction, before the electronic consent process:

*“For the purposes of this survey, PANS is considered to be Pediatric Acute-Onset Neuropsychiatric Syndrome, which is a disorder in which children experience a sudden and severe onset of obsessive-compulsive thoughts and behaviors along with other symptoms that are thought to be precipitated by an infection, environmental trigger, or metabolic disorder. The disorder is described by Swedo and colleagues (2012) and descriptions can be found here http://intramural.nimh.nih.gov/pdn/PANDAS-to-PANS2012.pdf. We consider PANS to include PANDAS, which is Pediatric Acute-Onset Neuropsychiatric Disorder Associated with Strep and PITAND, which is Pediatric Infection-Triggered Autoimmune Neuropsychiatric Disorder. Therefore, when we use the term “PANS” we mean PANS, PANDAS*, *and/or PITAND.”*

At the end of the consent form, potential participants were asked to click on one of two selections (“I agree to participate” or “I do not agree to participate.”) and were informed that clicking on the first selection indicated consent to participate in the research study. Participants were not compensated for participating.

Predefined logic checks were performed on data before analysis to “clean” the sample of records with illogical or incomplete information that called the quality of the reporting into question and/or that rendered the data uninterpretable. Through this process, we arrived at a sample of 698 surveys (derived from the 753 surveys submitted) considered to have complete and reliable information.

All data analyses were performed using the JMP^®^ statistical program. For treatment response questions, respondents were asked to classify response to treatment as “very effective,” “somewhat effective,” “not very effective,” “effective at first, then lost effect over time,” or “don't know/can't recall”; “don't know/can't recall” responses were then excluded from analyses of treatment effect. For treatment dose questions (relating to antibiotics, anti-inflammatories, and psychotropic medications), respondents were asked to classify doses as “high dose,” “regular dose,” or “prophylactic (low) dose,” or could state “don't know”; “don't know” responses were then excluded from analyses relating to dose.

## Results

### Demographics and clinical characteristics

Six hundred and ninety-eight patients were represented in the study sample. Ninety-five percent of surveys were completed by mothers of minor patients; 4% by fathers; and <1% by each of other primary caregivers and patients themselves (ages 18 and over only). Demographic and clinical characteristics of the patients are presented in detail elsewhere (Calaprice et al. 2017). Briefly, patients ranged in age from under 2 to 38 years old, with a median age of 11, and 80% between ages 7 and 17. Median age at onset of PANS symptoms was 7 years, and median age at diagnosis was 9 years. Sixty-four percent of the patients were male, and 34% female (the remainder did not report sex). Ninety-two percent of the patients resided in the United States; 45 states and the District of Columbia were represented.

Thirty-four percent of the patients (*N* = 227 of the 666 who answered the question) were reported to have immune deficiencies (*N* = 108), IgG levels in the low-normal range (*N* = 94), or low white counts (*N* = 25). Two hundred and four reported having apparently healthy immunity, and for 235, either no testing of immune sufficiency had been performed or the respondents did not know the results. Of those reporting immune deficiencies, nearly all were able to identify one or more specific laboratory values that were below the normal range. Forty-six reported total IgG below normal limits (BLN); 23 reported one or more IgG subclass deficiencies (IgG1: 9; IgG2: 13; IgG3: 7; IgG4: 14); 5 reported an inadequate response to pneumococcal vaccine with total IgG and IgG subclasses within normal limits (indicating Specific Antibody Deficiency); and 27 reported IgA BLN, although only 9 reported Selective IgA deficiency without concurrent deficiencies of IgG.

### Onset, infectious agents and antibiotic treatment

Disease onset was sudden, defined as reaching a concerning level within 3 days either from a starting point of no symptoms (for 64%) or in the context of existing symptoms or developmental issues (for 24%), for 88% of patients. For 94% of patients whose caregivers could recall (*N* = 623), this initial episode was associated with a confirmed (70%) or suspected (24%) infection, most commonly GAS (54% confirmed [*N* = 314], 8% suspected [*N* = 49]), although several other infectious agents were also reported and the presence of multiple infections was not uncommon [for details see Calaprice et al. (2017)]. Recurrences of PANS episodes, which occurred in the vast majority of patients, were also frequently associated with infections, with GAS again most common, although again, a variety of inciting infectious agents was reported.

To treat the PANS-associated infections, 97% of patients (*N* = 675) were reported to have been treated with short (defined by survey as fewer than 30 days; *N* = 503) and/or long (defined by survey as more than 30 days; *N* = 502) courses of antibiotics, most often amoxicillin, azithromycin, and/or amoxicillin–clavulanate (Augmentin^®^, Tables 1 and 2). Although amoxicillin was prescribed most commonly, it was least likely to be considered “very effective” or “somewhat effective” by participants in mitigating PANS symptoms. Of the antibiotics prescribed for short-term use (<30 days), clindamycin and metronidazole were most likely to be rated “very effective,” and of those prescribed for longer-term use (>30 days), amoxicillin–clavulanate and “other” antibiotics were most likely to be rated “very effective.” For most antibiotics, courses of >30 days were more likely than courses of fewer than 30 days to have been considered “very effective.” It should be noted, however, that approximately one-quarter of those receiving short courses were discontinued for lack of efficacy, so the greater response rate among those receiving longer courses could be due in part to a washing out of nonresponders before 30 days.

**Table 1.** Frequency of Use, Participant-Reported Effectiveness, and Reasons for Discontinuation for Short Courses (<30 Days) of Antibiotic Treatment in Controlling PANS Symptoms

		*Treatment effect*				*Reasons for discontinuation*^a^				
	N *reporting treatment effect*	*Very effective % (*N*)*	*Somewhat effective % (*N*)*	N *reporting current status*	N *reporting on discontinuation*	*Lack of efficacy % (*N*)*	*No longer perceived a need % (*N*)*	*Tolerability % (*N*)*	*Practitioner would no longer prescribe % (*N*)*	*Other % (*N*)*
Amoxicillin	235	20 (47)	26 (62)	217	204	28 (61)	21 (45)	5 (10)	31 (68)	9 (20)
Azithromycin	216	26 (56)	35 (76)	210	169	23 (49)	19 (40)	3 (7)	23 (48)	12 (25)
Amoxicillin clavulanate	184	30 (56)	32 (59)	171	151	22 (38)	18 (30)	9 (16)	26 (44)	13 (23)
Cefdinir	105	26 (27)	37 (39)	104	97	29 (30)	13 (14)	12 (12)	28 (29)	12 (12)
Clindamycin	80	41 (33)	20 (16)	86	78	22 (19)	22 (19)	3 (3)	35 (30)	8 (7)
Penicillin	67	18 (12)	36 (24)	64	57	25 (16)	14 (9)	8 (5)	33 (21)	9 (6)
Other cephalosporin	36	8 (3)	47 (17)	34	32	24 (8)	18 (6)	15 (5)	29 (10)	9 (3)
Doxycycline	21	14 (3)	48 (10)	23	20	17 (4)	22 (5)	22 (5)	22 (5)	4 (1)
Metronidazole	13	54 (7)	31 (4)	14	12	0 (0)	50 (7)	0 (0)	29 (4)	7 (1)
Other antibiotics	55	20 (11)	40 (22)	56	47	13 (7)	20 (11)	9 (5)	25 (14)	18 (10)

^a^ For “reasons for discontinuation” calculations, percentages use the total number reporting “current status” as the denominator.

PANS, pediatric acute-onset neuropsychiatric syndrome.

**Table 2.** Frequency of Use, Participant-Reported Effectiveness, and Reasons for Discontinuation for Long Courses (>30 Days) of Antibiotic Treatment in Controlling PANS Symptoms

		*Treatment effect*				*Reasons for discontinuation*^a^				
	N *reporting treatment effect*	*Very effective % (*N*)*	*Somewhat effective % (*N*)*	N *reporting current status*	N *reporting on discontinuation*	*Lack of efficacy % (*N*)*	*No longer perceived a need % (*N*)*	*Tolerability % (*N*)*	*Practitioner would no longer prescribe % (*N*)*	*Other % (*N*)*
Amoxicillin	129	27 (35)	30 (39)	123	89	31 (38)	16 (20)	8 (10)	10 (12)	7 (9)
Azithromycin	209	39 (81)	37 (77)	210	127	20 (41)	18 (38)	4 (9)	8 (16)	11 (23)
Amoxicillin clavulanate	196	52 (102)	26 (50)	194	96	8 (15)	18 (34)	6 (12)	8 (15)	10 (20)
Cefdinir	65	23 (15)	40 (26)	60	44	25 (15)	20 (12)	7 (4)	5 (3)	17 (10)
Clindamycin	44	32 (14)	30 (13)	49	35	20 (10)	18 (9)	4 (2)	22 (11)	6 (3)
Penicillin	76	39 (30)	32 (24)	73	42	19 (14)	19 (14)	4 (3)	4 (3)	11 (8)
Other cephalosporin	25	40 (10)	32 (8)	26	14	19 (5)	12 (3)	12 (3)	8 (2)	4 (1)
Doxycycline	25	44 (11)	44 (11)	26	16	8 (2)	23 (6)	12 (3)	12 (3)	8 (2)
Metronidazole	8	25 (2)	50 (4)	7	6	0 (0)	43 (3)	0 (0)	14 (1)	29 (2)
Other antibiotics	57	51 (29)	32 (18)	61	25	3 (2)	11 (7)	7 (4)	10 (6)	8 (6)

^a^ For “reasons for discontinuation” calculations, percentages use the total number reporting “current status” as the denominator.

Both short (<30 days) and long (>30 days) courses of antibiotic treatment were generally well tolerated by PANS patients, with fewer than 10% of those who received the most commonly prescribed antibiotics discontinuing because of side effects (Tables 1 and 2). Doxycycline (22%) and other cephalosporins (15%) were most likely to be discontinued because of side effects during the first 30 days of treatment. The most common reason that patients discontinued antibiotic treatment within 30 days of initiation was that after the initial course of therapy was finished, the healthcare practitioner would no longer continue to prescribe. Of the antibiotic courses greater than 30 days, amoxicillin was most likely to have been discontinued for lack of efficacy (31%), whereas amoxicillin–clavulanate (8%), doxycycline (8%), other antibiotics (8%), and metronidazole (0%) were least likely to have been discontinued for this reason (Table 2; these were also least likely to have been discontinued for lack of efficacy in patients receiving courses of <30 days, Table 1). Other reasons for discontinuing antibiotic therapy included the expense, and moving from one antibiotic to another as part of a rotational strategy or as new infectious agents were identified over time.

Table 3 presents antibiotic response data separately for each of the immunodeficient (*N* = 108) and “apparently healthy immunity” (*N* = 204) subsets of the sample. (To reduce uncertainty in interpretation, patients reported as having “low-normal” IgG levels or other possible immune abnormalities [e.g. low white counts], or for whom immune health was unknown, were excluded from this analysis.) There was a trend whereby both short and long courses of most antibiotics were “very effective” in controlling PANS symptoms more often for patients with healthy immunity than for those with immune deficiencies. This was particularly true for antibiotics with a relatively narrow spectrum of activity, including amoxicillin and penicillin; for amoxicillin–clavulanate, in contrast, immunodeficient patients and those with healthy immunity showed similar rates of response. Thus, although the probability of a very effective response was similar across many antibiotics for patients with healthy immunity, response rates appeared to differ across antibiotics for patients with immune deficiencies.

**Table 3.** Percent of Patients with Healthy Immunity Versus Immune Deficiency for Which Most Commonly Prescribed Antibiotics Were Considered “Very Effective” by Participants

	*<30-day course*				*>30-day course*			
	*Healthy immunity*		*Immune deficient*		*Healthy immunity*		*Immune deficient*	
	N *reporting use*	*Very effective % (*N*)*	N *reporting use*	*Very effective % (*N*)*	N *reporting use*	*Very effective % (*N*)*	N *reporting use*	*Very effective % (*N*)*
Amoxicillin	71	27 (19)	31	13 (4)	36	42 (15)	25	16 (4)
Azithromycin	54	28 (15)	33	21 (7)	53	51 (27)	48	33 (16)^a^
Amoxicillin/clavulanate	60	28 (17)	25	32 (8)	57	61 (35)	35	54 (19)
Cefdinir	25	28 (7)	19	26 (5)	15	20 (3)	16	13 (2)
Clindamycin	17	59 (10)	17	35 (6)	11	27 (3)	11	27 (3)
Penicillin	17	18 (3)	9	11 (1)	20	55 (11)	9	22 (2)^+^

^a^ Significant difference from those with healthy immunity, DF = 1, *χ*^2^ = 8.6, *p* = 0.0351.

### Anti-inflammatory medications

Four hundred and thirty-seven patients had utilized anti-inflammatory medications in attempt to relieve PANS symptoms (Table 4). Ibuprofen was reportedly used by many patients (43%) to manage PANS symptoms, and for 80% was at least somewhat effective (Table 4). A dose–response relationship was apparent: among those who used “high doses” (*N* = 42), 43% found ibuprofen to be “very effective” at managing PANS symptoms and 93% found it to be *at least* “somewhat effective”; in comparison, 20% of those who used each of “regular doses” (*N* = 228) and “low doses” (*N* = 30) found it to be “very effective,” and 79% and 77%, respectively, found it at least somewhat effective (*χ*^2^ = 19, *p* < 0.03). Only 18 patients (6%) discontinued ibuprofen for reasons related to tolerability.

**Table 4.** Frequency of Reported Use, Participant-Reported Effectiveness, and Reasons for Discontinuation of Anti-Inflammatory Medications

		*Treatment effect % (*N*)*				*Reasons for discontinuation*^a^*% (*N*)*				
*Medication*	N *reporting treatment effect*	*Very effective*	*Somewhat effective*	N *reporting on current status*	N *reporting discontinuation*	*Lack of efficacy % (*N*)*	*No longer perceived a need % (*N*)*	*Tolerability % (*N*)*	*Practitioner would no longer prescribe % (*N*)*	*Other % (*N*)*
Ibuprofen	302	23 (69)	57 (172)	289	155	10 (29)	21 (61)	6 (18)	2 (6)	14 (41)
Steroid taper, <14 days	154	49 (76)	23 (35)	147	144	7 (11)	22 (33)	17 (25)	37 (55)	14 (20)
Steroid taper, >14 days	72	54 (39)	21 (15)	68	62	3 (2)	24 (16)	24 (16)	26 (18)	15 (10)
Allergy medicines, <30 days	72	14 (10)	56 (40)	71	42	17 (12)	23 (16)	8 (6)	4 (3)	7 (5)
Allergy medicines, >30 days	101	20 (20)	58 (59)	101	29	9 (9)	8 (8)	6 (6)	1 (1)	5 (5)
Other anti-inflammatory medications	53	38 (20)	55 (29)	58	25	3 (2)	16 (9)	7 (4)	5 (3)	12 (7)

^a^ For “reasons for discontinuation” calculations, percentages use the total number reporting “current status” as the denominator.

Steroid tapers were very effective for approximately half of patients, and at least somewhat effective for approximately three quarters. Allergy medications (including cetirizine [Zyrtec], Mometasone Furoate [Nasonex], and diphenhydramine [Benadryl]), when used for >30 days, had a similar efficacy and tolerability profile to ibuprofen; they were very effective for only a minority (20%) of patients, but at least somewhat effective for most (78%), and only 7% (*N* = 12) discontinued for reasons related to tolerability.

In the open-ended comments section regarding medications, celecoxib (Celebrex) and naproxen were each described as being very effective for two patients. Naproxen was described as being more effective than ibuprofen, but for one patient was discontinued because of gastrointestinal side effects.

### Intravenous immunoglobulin

Of the patients who reported on both immune status and use of IVIG (*N* = 656), IVIG use was reported for 206 patients (31%), but only 191 reported on its therapeutic impact (Table 5). On the whole, this therapy was reported to be very effective for 49% of patients, somewhat effective for 25%, not very effective for 11%, and effective at first, but not enduringly so, for 16% (Table 5). IVIG was most likely to have been prescribed for patients with IgG deficiencies (59% received this treatment), and was also most likely to be very effective or somewhat effective (90%), and enduringly effective (only 5% report effectiveness lost over time), in this patient subset (difference in treatment effect between IgG-deficient and all other patients, excluding those for whom immune status was unknown: *χ*^2^ = 8.0, DF = 3, *p* < 0.05). With respect to treatment response, patients with low-normal IgG levels appeared more similar to those with healthy immunity than they were to those with true IgG deficiencies.

**Table 5.** Frequency of Use and Effectiveness of IVIG Treatment, by Treatment Regimen and Immune Status of Patients (*N* = 656 Patients Who Reported Both Immune Status and Whether IVIG Was Used)

				*Treatment effect, % (*N*)*			
*Immune status before IVIG*	*IVIG treatment program*	*Dose (g/kg)*	N *reporting use and effect*	*Very effective*	*Somewhat effective*	*Not very effective*	*Effective at first, then lost effect*
Apparently healthy immunity (*N* = 202)	Recurrent use, as required by symptom course	<1.2	1		100 (1)		
		1.2–1.9	10	60 (6)	30 (3)		10 (1)
		≥2	7	14 (1)	29 (2)	14 (1)	43 (3)
		Unknown	3	100 (3)			
	Regularly scheduled treatment, every 2–5 weeks	<0.8	1			100 (1)	
		0.8–1.1	2	100 (2)			
		1.2–2.0	2			50 (1)	50 (1)
		Unknown	1		100 (1)		
	Regularly scheduled treatment, every 6–12 weeks	1.2–2.0	1			100 (1)	
	Single treatment	0.8–1.1	3	67 (2)		33 (1)	
		1.2–1.9	6	50 (3)	17 (1)	17 (1)	17 (1)
		2	10	60 (6)	10 (1)	20 (2)	10 (1)
		Unknown	8	88 (7)	13 (1)		
	Total		55 (27%)	55% (30)	18% (10)	15% (8)	13% (7)
Low-normal IgG (*N* = 93)	Recurrent use, as required by symptom course	1.2–1.9	2	100 (2)			
		≥2	4	25 (1)		50 (2)	25 (1)
		Unknown	2	100 (2)			
	Regularly scheduled treatment, every 2–5 weeks	<0.8	1				100 (1)
		0.8–1.1	1	100 (1)			
		1.2–2.0	3	67 (2)		33 (1)	
	Regularly scheduled treatment, every 6–12 weeks	0.8–1.1	2		100 (2)		
		1.2–2.0	4	75 (3)			25 (1)
	Single treatment	0.8–1.1	2		50 (1)	50 (1)	
		1.2–1.9	7	43 (3)		14 (1)	43 (3)
		2	10	60 (6)			40 (4)
		Unknown	1		100 (1)		
	Total		41 (44%)	49 (20)	10 (4)	12 (5)	29 (12)
IgG deficiencies^a^ (*N* = 62)	Recurrent use, as required by symptom course	1.2–1.9	4	75 (3)	25 (1)		
		2 or more	6	67 (4)			33 (2)
	Regularly scheduled treatment, every 2–5 weeks	<0.8	6	43 (3)	57 (4)		
		0.8–1.1	4	100 (4)			
		1.2–2.0	1		100 (1)		
	Regularly scheduled treatment, every 6–12 weeks	1.2–2.0	3	33 (1)	33 (1)	33 (1)	
	Single treatment	2	2	50 (1)	50 (1)		
		Unknown	2	50 (1)	50 (1)		
	Other regimen	Various	7	57 (4)	29 (2)	14 (1)	
	Regimen not reported	Not reported	1	100 (1)			
	Total		37 (59%)	60 (22)	30 (11)	5 (2)	5 (2)
IgA BNL without IgG deficiency (*N* = 9)	Single treatment	1.2–1.9	1		100 (1)		
		2	1		100 (1)		
	Total		2 (22%)		100 (2)		
Low white count (*N* = 25)	Recurrent use, as required by symptom course	1.2–1.9	1				100 (1)
		2 or more	1	100 (1)			
	Regularly scheduled treatment, every 2–5 weeks	0.8–1.1	2		100 (2)		
		Unknown dose	1				100 (1)
	Single treatment	2	3	33 (1)	33 (1)	33 (1)	
	Total		8 (32%)	25 (2)	38 (3)	13 (1)	25 (2)
Immune deficiency, unspecified (*N* = 35)	Recurrent use, as required by symptom course	1.2–1.9	1			100 (1)	
		2 or more	1		100 (1)		
		Unknown dose	1		100 (1)		
	Regularly scheduled treatment, every 2–5 weeks	<0.8	1		100 (1)		
	Regularly scheduled treatment, every 6–12 weeks	1.2–2.0	8	63 (5)	25 (2)	13 (1)	
		Unknown dose	1	100 (1)			
	Single treatment	1.2–1.9	1			100 (1)	
		2 or more	1		100 (1)		
		Unknown dose	1	100 (1)			
	Total		16 (46%)	44 (7)	38 (6)	19 (3)	
Not tested (*N* = 230)	Recurrent use, as required by symptom course	1.2–1.9	2	50 (1)			50 (1)
		2 or more	7	29 (2)	14 (1)		57 (4)
		Unknown dose	1	100 (1)			
	Regularly scheduled treatment, every 2–5 weeks	0.8–1.1	1		100 (1)		
		1.2–2.0	1	100 (1)			
		Unknown dose	1				100 (1)
	Single treatment	0.8–1.1	1			100 (1)	
		1.2–1.9	3	33 (1)		33 (1)	33 (1)
		2	9	33 (3)	67 (6)		
		Unknown	4	25 (1)	75 (3)		
	Other regimen	Various	2	100 (2)			
	Total		32 (14%)	38 (12)	34 (11)	6 (2)	22 (7)
Grand total			191	49 (93)	25 (47)	11 (21)	16 (30)

^a^ Includes total IgG below normal limit (BNL), any subclass BNL, or specific antibody deficiency (inadequate response to pneumococcal vaccine).

BNL, below normal limit; IVIG, intravenous immunoglobulin.

Not surprisingly, IVIG regimens varied considerably according to a patient's immune status (Table 5). Patients with IgG deficiencies were most often prescribed recurrent, scheduled treatments, whereas those in all other specific immunocompetence categories (i.e., excluding “unspecified,” “not tested,” or “no response”) were most often prescribed single doses. All IgG-deficient patients who received IVIG every 2–5 weeks found it to be at least somewhat effective, and most (58%) found it very effective. Although N's were small, the optimum dose at this frequency appeared to be between 0.8 and 1.1 g/kg (*N* = 4; 100% very effective). Interestingly, all patients in other immune health categories also found regularly scheduled doses of 0.8–1.1 k/kg to be at least somewhat, and often (38%) very, effective in a sustained manner (*N* = 8). In contrast, both initial treatment failure (not very effective:13%) and regression over time after an initial response (22%) were more frequent with higher doses of IVIG when used in a regular or recurrent fashion, which was significantly different from the finding of 0 initial treatment failure and 0 regression over time found with the 0.8–1.1 g/kg regimen (*N* = 82; *χ*^2^ = 11.7, DF = 3, *p* < 0.01).

### Plasmapheresis

Twenty-five patients in this study had received plasmapheresis (4%). When asked to describe the treatment outcome in an open-ended manner (this question was not asked in a multiple-choice fashion), 19 respondents were able to describe the outcome of treatment. Most of these (*N* = 15; 79%) reported a positive initial response to treatment, but many of these (*N* = 9; 60%), specified that the positive effects were not enduring, with symptoms recurring particularly once the patient was exposed to another infection.

### Psychotropic medications

Many patients (*N* = 379) had received psychotropic medications to treat PANS symptoms, although only approximately half found each class of such medications to have been somewhat effective or very effective (Table 6). SSRIs were prescribed most commonly, although these were very effective at managing PANS symptoms for only 17% of patients, and somewhat effective for an additional 27%; response rates were similar for non-SSRI antidepressants. Likewise, ADHD, antipsychotic, and anxiolytic medications were at least somewhat effective for approximately half of the patients to whom they were prescribed, but were very effective for only the minority. Mood-stabilizing medications were the least likely of the psychotropic medications to be somewhat or very effective.

**Table 6.** Frequency of Reported Use, Participant-Reported Effectiveness, and Reasons for Discontinuation of Psychopharmacological Medications

		*Treatment effect % (*N*)*				*Reasons for discontinuation % (*N*)*				
*Medication*	N *reporting treatment effect*	*Very effective*	*Somewhat effective*	N *reporting on current status*	N *reporting discontinuation*	*Lack of efficacy % (*N*)*	*No longer perceived a need % (*N*)*	*Tolerability % (*N*)*	*Practitioner would no longer prescribe % (*N*)*	*Other % (*N*)*
SSRIs (e.g., fluoxetine, sertraline, etc.)	265	17 (46)	27 (71)	260	136	20 (52)	6 (15)	25 (64)	1 (2)	1 (3)
Other antidepressants	60	15 (9)	28 (17)	57	37	28 (16)	7 (4)	21 (12)	4 (2)	5 (3)
ADHD medications (e.g., methylphenidate, etc.)	114	20 (23)	34 (39)	112	61	18 (20)	1 (1)	32 (36)	1 (1)	3 (3)
Antipsychotic medications	95	13 (12)	38 (36)	97	58	20 (19)	15 (15)	22 (21)	1 (1)	2 (2)
Anxiolytic medications (e.g., diazepam, lorazepam, etc.)	84	20 (17)	26 (22)	83	57	25 (21)	12 (10)	28 (23)	1 (1)	2 (2)
Mood-stabilizing/anticonvulsant medications (e.g., valproate, carbamazepine, lithium, etc.)	63	13 (8)	25 (16)	63	41	16 (10)	13 (8)	30 (19)	2 (1)	5 (3)
Other neuroactive medications	34	26 (9)	53 (18)	32	15	16 (5)	9 (3)	16 (5)	3 (1)	3 (1)

SSRIs, selective serotonin reuptake inhibitors; ADHD, attention deficit/hyperactivity disorder.

Patients who had received psychotropic medications had discontinued them 65% of the time (Table 6), and only 14% of these discontinued because they no longer perceived a need. In contrast to the situation for antibiotic treatment, psychotropic mediations had most often been discontinued because of side effects (21%–32% depending on the medication), although rates of discontinuation for lack of efficacy were not far behind (16%–28%). Although the survey did not request that participants describe the specific side effects that led to discontinuation, there were 10 spontaneous reports (out of the 64 patients who discontinued for tolerability reasons), of significant or even dramatic worsening of psychiatric symptoms, including mania, psychosis, suicidality, aggression/violence, hyperactivity, and agitation, with SSRI treatment. Weight changes, somnolence, and blunted affect also contributed to discontinuation of psychotropic medications for some subjects.

### Psychotherapy

Many of the patients represented in this survey had received some form of psychotherapy for their PANS symptoms (*n* = 473; Table 7). No psychotherapeutic approach was reported to have been very effective for most patients, but many found such approaches at least somewhat effective. The highest rate of reported effectiveness was for CBT with exposure/response prevention, with over two-thirds of those who had received this or were currently receiving this reporting that it was very or somewhat effective. In the section allowing for open-ended comments, a few respondents mentioned that medical therapies were required to “open the door” for successful treatment with CBT.

**Table 7.** Frequency of Use and Effectiveness of Psychotherapy

	*Received in the past*			*Currently receiving*		
		*Treatment effect % (*N*)*			*Treatment effect % (*N*)*	
*Type of psychotherapy*	N *reporting treatment effect*	*Very effective*	*Somewhat effective*	N *reporting treatment effect*	*Very effective*	*Somewhat effective*
CBT	159	21 (34)	33 (53)	139	17 (23)	56 (78)
CBT +ERP	71	39 (28)	28 (20)	44	27 (12)	43 (19)
Habit reversal therapy	13	8 (1)	23 (3)	9	11 (1)	33 (3)
Behavior management	48	4 (2)	46 (22)	62	13 (8)	63 (39)
Counseling	143	8 (12)	36 (51)	124	8 (10)	59 (73)
Other	22	9 (2)	41 (9)	27	26 (7)	52 (14)

CBT, cognitive behavioral therapy; ERP, exposure response prevention.

### Complementary and alternative medicines

More than half of the families (*N* = 352) reported finding some type of CAM helpful, with over 75 different CAM identified in the open-ended questions. The responses were reviewed and coded as vitamins, supplements (nonvitamin dietary supplements), diets, and therapies, as noted in Table 8. Of these, the most frequently used treatments were supplements, with 200 families reporting favorable outcomes from at least one supplement. About 25% (*N* = 85) of the families using CAM found probiotics to be beneficial and 22% (*N* = 72) found benefit from fish oil or other omega 3 supplements. Vitamins were found to be helpful by 122 families (35%), with vitamin D most commonly cited as beneficial. A decidedly smaller number of families found diets (*N* = 48) and other therapies (*N* = 44) to be of benefit, with gluten-free being the most common diet (*N* = 30) and homeopathy the most common therapy (*N* = 27). Interestingly, only 83 families identified one or more CAM that was not found to be of benefit (Table 8). Of those, probiotics were not helpful for 10 and homeopathy was not helpful for 11, which aligns with the high use of these CAM among patients. Other CAM therapies were reported to not be beneficial by small numbers of participants.

**Table 8.** CAM that Participants Found Helpful and Unhelpful

	*N report use*	*Helpful % (N)*	*Not Helpful % (N)*		*N report use*	*Helpful % (N)*	*Not Helpful % (N)*
*Misc. Supplements*				*Vitamins/Minerals*			
Probiotic	95	89 (85)	11 (10)	Vitamin D	59	97 (57)	3 (2)
Fish Oil/Omega 3	78	92 (72)	8 (6)	B vitamins/inositol	46	93 (43)	7 (3)
Turmeric/Curcumin	32	94 (30)	6 (2)	Vitamin C	21	100 (21)	0 (0)
N-Acetyl Cysteine	22	86 (19)	14 (3)	Zinc	12	100 (12)	0 (0)
Melatonin	17	100 (17)	0 (0)	Magnesium	32	94 (30)	6 (2)
Essential oils	6	100 (6)	0 (0)	Other Vitamin^*^	56	95 (53)	5 (3)
Oregano Oil	5	100 (5)	0 (0)				

*Diets*					*Other Therapies*		
Gluten Free	35	86 (30)	14 (5)	Homeopathy	38	71 (27)	29 (11)
Dairy/Casein Free	20	85 (17)	15 (3)	Acupressure/puncture	9	78 (7)	22 (2)
Reduced sugar	6	100 (6)	0 (0)	Chiropractic	6	83 (5)	17 (1)
Other Dietary changes^*^	38	74 (28)	16 (10)	Other Therapy^*^	88	64 (56)	36 (32)

^*^ Compilation of CAM for which N<5 each.

CAM, complementary and alternative medicine.

## Discussion

Although the importance of effective intervention in impacting both the short-term and the long-term course of PANS has been noted (Calaprice et al. 2017), knowledge about the efficacy and tolerability of the variety of interventions being used has been limited by small sample sizes, restrictive eligibility criteria for clinical studies, and other methodological challenges. While studies of several individual interventions appear in the literature, this is the first study to report on the range and frequency of use of different interventions in a large community sample of patients with PANS, as well as on the patient- or caregiver-reported effectiveness and tolerability of each.

This study supports the current practice of antibiotic therapy as first-line treatment. The majority of patients represented in this study had received antibiotic treatment for PANS; most had found this treatment at least somewhat effective in reducing PANS symptoms, and well tolerated. Treatment with broad-spectrum antibiotics and long courses was reported to be most successful, consistent with the high prevalence of infections and relatively high rates of immune deficits that characterize this population. Aggressive treatment to eradicate infection appeared to be particularly important in the subset of patients with the weakest immune systems. It was of interest that the use of narrow-spectrum antibiotics, such as amoxicillin and penicillin were less effective. Recurrences with GAS and incomplete eradication of GAS are reported to be higher with these therapies (Brook and Foote 2005; Brook and Gober, 2006), possibly due to the presence of beta-lactamase-producing bacteria in the oral flora. Other antimicrobials, such as amoxicillin–clavulanate and cefdinir, offer protection against beta-lactamase-producing organisms. The potential for certain antimicrobials to possess neurochemical or immune-modulating properties is also a consideration (Obregon et al. 2012) The need for sufficiently aggressive antibiotic treatment from the first PANS presentation is particularly important given that, as reported previously (Calaprice et al. 2017), resolution of the inciting infection with the initial course of antibiotic treatment is achieved for only 59% of patients, and recurrence of PANS appears to be significantly more probable for patients for whom the infection associated with the initial PANS episode does not resolve completely with antibiotic treatment. Controlled studies are needed to better prove and delineate the role of antimicrobial treatment at onset of PANS and throughout the course of PANS.

IVIG therapy was reported to have been beneficial in reducing PANS symptoms for many patients, but was most beneficial, and most enduringly beneficial, for the subset with IgG deficiencies. This trend supports the idea that the primary role of IVIG therapy in PANS may be to support the immune system in eradicating and preventing infection. However, the observation that the most effective doses appear to be somewhat higher than seen in typical treatment of immune deficiency, and that benefit is also observed in immunologically healthy patients, suggest an additional role in suppression of the autoimmune mechanism. As might be expected, the beneficial effect of IVIG in PANS generally appears to be temporary in the absence of ongoing treatment, as both mechanisms rely on sufficient levels of IgG in the bloodstream. That PANS represents a continuous vulnerability with repeated infection, and that acute therapeutic suppression of the autoimmune mechanism may therefore only have a temporary benefit, are also suggested by data regarding plasmapheresis treatment in this population.

New in this study is the suggestion that the highest doses of IVIG (2 g/kg) may not be as enduringly effective as more moderate doses. Although the number of patients represented in this part of the study are small and caution must be exercised in interpretation, it is possible that somewhat lower doses are sufficient to suppress the autoimmune mechanism in this population, and that, as has been reported elsewhere, the half-life of IgG decreases with increasing dose (Schiff and Rudd 1986). Trials examining the timing and dose of IVIG as well as the characteristics of those achieving benefit would be of interest.

The use of plasmapheresis in this study was limited to only 25 patients, compared with 193 who received IVIG intervention. Latimer et al. (2015) report on 40 patients at Georgetown University Hospital, and note that plasmapheresis is reserved for patients who have had little success with antibiotics and who demonstrate aggressive, violent behavior, are a danger to themselves or others, have severe food restriction, and/or lack of response to oral steroids and IVIG. The patients at Georgetown all found relief with plasmapheresis, but the relief was generally not sustained when reinfection occurred, which was also seen in our sample. This reinforces the use of plasmapheresis for severe cases with the recognition that ongoing antibiotic or immune-modulating therapies should be considered.

The autoimmune pathophysiology of PANS may result not only from the adverse effects of neuroactive autoantibodies, but also from elevations of inflammatory mediators such as neuroactive cytokines. A role for inflammatory mediators in psychiatric illness has been identified in both children (Mitchell and Goldstein 2014) and adults (Baumeister et al. 2014). In addition to their direct action, inflammatory mediators have also been posited, in PANS, to create a breach in the blood–brain barrier that permits neuroactive autoantibodies to reach neuronal tissue (Swedo et al. 2012). That such mechanisms may indeed be critical in PANS is suggested by the efficacy of anti-inflammatory therapies, most commonly ibuprofen and steroids, in attenuating PANS symptoms among patients in this study. Although treatment with other anti-inflammatories, such as naproxen and celecoxib, appears to be rare in the PANS population, the positive reports collected in this survey suggest that this anti-inflammatory treatment strategy may deserve further study (Mahony et al. 2017).

A significant fraction of the survey patients had been prescribed psychotropic medications, particularly including SSRIs, but the therapeutic results reported were mixed. As was seen in previous research (Murphy et al. 2006), SSRIs were poorly tolerated by many patients in this study at standard doses, although some were able to tolerate lower doses. Thus, this research again suggests that it may be advisable to initiate therapy with low doses in those presenting with PANS and titrate slowly upward as tolerated. The mechanism whereby SSRIs may cause exacerbation of psychiatric symptoms in PANS patients is not known, but may be consistent with a general neurological hypersensitivity, or with the high rates of co-occurring psychiatric symptoms, younger age of patient, etc., as has been reported previously (Murphy et al. 2006).

CBT, particularly when combined with exposure/response prevention (ERP), appeared to be at least somewhat effective for most patients. Some respondents commented that benefits were only achieved once the medical aspects of the disease were sufficiently well managed to allow children to fully participate and respond. This parallels the treatment approach of non-PANS OCD in that youth with severe OCD may better tolerate CBT once the anxiety is partially alleviated with SSRI therapy. Given the lack of pharmaceutical tolerability concerns, along with the moderate rate of efficacy in this sample and widespread utility in non-PANS OCD, use of CBT with ERP should be considered in PANS patients who are receiving medical therapies with adequate response, but with residual symptoms that could benefit from this additional approach.

Although this study did not investigate CAM approaches in a systematic manner, the high reported rate of CAM use and high perception of benefit from CAM suggests that further examination of these options may be worthwhile. The available literature supports the use of omega 3's as adjunct therapies for ADHD (Bloch and Qawasmi 2011), depression (Sarris et al. 2016), and emotional lability (Cooper et al. 2016). However, in virtually every case in this study the patients also required “standard” medical therapies to achieve adequate disease control. Thus, there was no indication in this study that PANS could be effectively managed exclusively with CAM approaches; in fact, it was noted by one parent that “alternative therapy delayed her [the patient] getting the proper treatment early.” Healthcare providers should be aware, however, that patients are likely using CAM and should ask about those therapies and provide guidance when making medical decisions.

This study possessed several limitations, including reliance on participant recall, lack of clinical confirmation of diagnosis, treatment dose and response, and reasons for treatment discontinuation. Furthermore, the survey design did not permit examination of the order in which treatments had been received and/or whether they had been prescribed individually or in combination, which could have influenced their perceived efficacy. The study may also have suffered a participation bias compared with the PANS population at large, since it is possible that the caregivers of patients with ongoing symptoms may have been more interested in or aware of the study opportunity than caregivers of patients in remission. Data collection was completed in 2014, and treatment practices may have changed since then, particularly in terms of the frequency of use of different approaches.

## Conclusion

Among the PANS patients represented in this study, relatively aggressive treatment courses targeted at eradicating infection and modulating the inflammatory response appeared to provide the best caregiver-reported therapeutic results, and to be generally well tolerated. Aggressive treatment of this type appeared to be particularly important for the significant subset of patients with poor immune function. Given its relative efficacy and tolerability, treatment targeting the inflammatory response may represent an underutilized approach in this population and deserves further study, particularly given the wide range of potent and targeted anti-inflammatory and cytokine-inhibiting therapies currently available. Of the treatment modalities typically used with non-PANS psychiatric disorders, both psychotherapeutic and psychopharmacological approaches were quite common in this PANS population as well. As with non-PANS OCD, CBT, particularly when enhanced with exposure response prevention and when performed in conjunction with medical approaches, was often at least somewhat effective. Similarly, psychopharmacological approaches provided benefits to some PANS patients in this study, although with poorer tolerability than is generally reported in the context of non-PANS psychiatric patients. It would be of interest in future studies to perform direct comparisons of the efficacy and tolerability of, and dosing/titration strategies for, these treatment approaches in youth with PANS and with non-PANS psychiatric presentations, not only to optimize treatment strategies but also because reactions to treatment may assist in differential diagnosis. This study highlights the need for further research on treatments for PANS, as well as the need to create standards of care both for the PANS population overall, and for important clinical subsets of patients therein.

## Clinical Significance

Data obtained in this study support the current practice of antibiotic therapy as first-line treatment, whereas at the same time underscoring the need for antibiotics to be of adequate therapeutic spectrum, and in courses of adequate length, to fully address infection, particularly in immunocompromised patients. IgG-deficient patients should be supported with regular IVIG therapy at sufficient doses; some patients with healthy immunity may benefit from IVIG treatment as well. Anti-inflammatory therapies, including nonprescription therapies are well tolerated, often effective, and relatively easily accessible by patients, and likely represent an underutilized treatment approach. Psychotropic medications, while useful in many patients, should be started and titrated conservatively. Cognitive behavioral therapy, particularly in conjunction with exposure/response prevention, is often helpful, and should always be considered in patients for whom PANS is under sufficient medical control to allow for full participation. Finally, it must be noted that as with any therapeutic intervention, appropriate application of the various treatments for PANS must rest heavily on clinician judgment based on the detailed history of the individual patient.

## Author Contributions

Dr. Calaprice is lead investigator, designed the study, was responsible for primary data analysis and interpretation of data, and is the principal writer of the article. Dr. Tona assisted in study design, managed data collection, and contributed to article writing and critical review of the article. Dr. Murphy assisted in study design and content, article writing, and critical review of the article.
